# The Role of Pathological Method and Clearance Definition for the Evaluation of Margin Status after Pancreatoduodenectomy for Periampullary Cancer. Results of a Multicenter Prospective Randomized Trial

**DOI:** 10.3390/cancers13092097

**Published:** 2021-04-26

**Authors:** Gennaro Nappo, Domenico Borzomati, Alessandro Zerbi, Paola Spaggiari, Ugo Boggi, Daniela Campani, Sławomir Mrowiec, Łukasz Liszka, Alessandro Coppola, Michela Amato, Tommasangelo Petitti, Fabio Vistoli, Marco Montorsi, Giuseppe Perrone, Roberto Coppola, Damiano Caputo

**Affiliations:** 1 Pancreatic Surgery Unit, Humanitas Clinical and Research Center—IRCCS, Via Manzoni 56, 20089 Rozzano, Italy; gennaro.nappo@humanitas.it (G.N.); alessandro.zerbi@humanitas.it (A.Z.); 2 Department of Surgery, Università Campus Bio-Medico di Roma, 00128 Rome, Italy; d.borzomati@unicampus.it (D.B.); r.coppola@unicampus.it (R.C.); d.caputo@unicampus.it (D.C.); 3 Department of Biomedical Sciences, Humanitas University, Via Rita Levi Montalcini 4, 20090 Milan, Italy; marco.montorsi@humanitas.it; 4 Pathology Unit, Humanitas Clinical and Research Center—IRCCS, Via Manzoni 56, 20089 Rozzano, Italy; paola.spaggiari@humanitas.it (P.S.); u.boggi@med.unipi.it (U.B.); f.vistoli@med.unipi.it (F.V.); 5 Division of General and Transplant Surgery, Azienda Ospedaliero-Universitaria Pisana, University of Pisa, 56126 Pisa, Italy; 6 Pathology Unit, Azienda Ospedaliero-Universitaria Pisana, University of Pisa, 56126 Pisa, Italy; d.campani@med.unipi.it; 7 Department of Gastrointestinal Surgery, Medical University of Silesia, 40-055 Katowice, Poland; smrowiec@sum.edu.pl; 8 Department of Pathomorphology and Molecular Diagnostics, Medical University of Silesia, 40-055 Katowice, Poland; lliszka@mp.pl; 9 Pathology Unit, Campus Bio-Medico University of Rome, 00128 Rome, Italy; m.amato@unicampus.it (M.A.); g.perrone@unicampus.it (G.P.); 10 Department of Public Health, Hygiene and Statistics, Campus Bio-Medico University of Rome, 00128 Rome, Italy; t.petitti@unicampus.it; 11 Department of Surgery, Humanitas Clinical and Research Center—IRCCS, Via Manzoni 56, 20089 Rozzano, Italy

**Keywords:** pancreatoduodenectomy, margin status, R1 resection, pathological evaluation, minimum clearance, microscopic residual tumor

## Abstract

**Simple Summary:**

There is no clear evidence on the most effective method of pathological analysis and clearance definition (0 vs. 1 mm) to define R1 resection after pancreatoduodenectomy (PD). However, several studies showed that the R1 resection is a poor prognostic factor in patients that have undergone PDs for periampullary cancers. In this randomized clinical trial, specimens were randomized with two pathological methods, the Leeds Pathology Protocol (LEEPP) or the conventional method adopted before the study. The 1 mm clearance is the most effective factor in determining R1 rate after PD but only when adopting the LEEP, the R1 resection represents a significant prognostic factor.

**Abstract:**

Background: There is extreme heterogeneity in the available literature on the determination of R1 resection rate after pancreatoduodenectomy (PD); consequently, its prognostic role is still debated. The aims of this multicenter randomized study were to evaluate the effect of sampling and clearance definition in determining R1 rate after PD for periampullary cancer and to assess the prognostic role of R1 resection. Methods: PD specimens were randomized to Leeds Pathology Protocol (LEEPP) (group A) or the conventional method adopted before the study (group B). R1 rate was determined by adopting 0- and 1-mm clearance; the association between R1, local recurrence (LR) and overall survival (OS) was also evaluated. Results. One-hundred-sixty-eight PD specimens were included. With 0 mm clearance, R1 rate was 26.2% and 20.2% for groups A and B, respectively; with 1 mm, R1 rate was 60.7% and 57.1%, respectively (*p* > 0.05). Only in group A was R1 found to be a significant prognostic factor: at 0 mm, median OS was 36 and 20 months for R0 and R1, respectively, while at 1 mm, median OS was not reached and 30 months. At multivariate analysis, R1 resection was found to be a significant prognostic factor independent of clearance definition only in the case of the adoption of LEEPP. Conclusions. The 1 mm clearance is the most effective factor in determining the R1 rate after PD. However, the pathological method is crucial to accurately evaluate its prognostic role: only R1 resections obtained with the adoption of LEEPP seem to significantly affect prognosis.

## 1. Introduction

The evaluation of margin status after pancreatoduodenectomy (PD) for periampullary tumors has been a widely debated topic over the past decade [1,2,3]. Before 2005, the reported microscopic involvement (R1) rate after PD was considerably lower, ranging from 20% to 30% [4,5]. However, in 2006, Verbeke et al. described a new method for pathological evaluation, the Leeds Pathology Protocol (LEEPP), which resulted in a significantly increased R1 rate of up to 70% [6].

When compared with traditional pathological methods, LEEPP includes some new concepts: the ‘circumferential margin’ (i.e., the entire surface of the PD specimen is examined during the pathological evaluation), the axial slicing of the PD specimen (as opposed to the bivalve slicing previously adopted), and a new definition of minimum clearance for defining R1 resection (1 vs. 0 mm) [7].

After the introduction of the LEEPP, several studies adopting this protocol were reported, confirming its impact on the R1 rate [8,9,10]. However, questions remain unanswered. In particular, if the LEEPP is adopted, is the higher R1 rate due only to the different adopted clearance or does the different sampling itself play a role? Other pathological protocols have also been described in the literature [11,12], but no consensus on which one should be adopted has been reached [13]. These heterogeneities mean that an interpretation of the available literature is difficult, especially for the evaluation of the prognostic role of R1 after PD, with some but not all studies reporting an impact of R1 on prognosis [14,15,16,17,18].

In this multicenter study, patients undergoing PD had their specimens randomized to the LEEPP method or a conventional method that was used before the study. Since the clearance is a potential confounding factor, R1 was defined in all enrolled patients as both 0- and 1-mm clearances. We were therefore able to determine the real impact of the pathological method itself on reported R1 rate and its prognostic value.

## 2. Materials and Methods

Patients undergoing PDs performed for pancreatic ductal adenocarcinoma (PDAC), distal cholangiocarcinoma (DC), and ampullary cancer (AC) were considered eligible. Exclusion criteria were previous pancreatic surgery, positive transection margins and R2 resection. Four medium-high volume pancreatic centers were involved.

The study was approved by the ethical committee of each participating center. Written informed consent was obtained before surgery. The study was registered at ClinicalTrials.gov (NCT03267966).

### 2.1. Randomization and Methods of Pathological Evaluation

**Figure 1:**
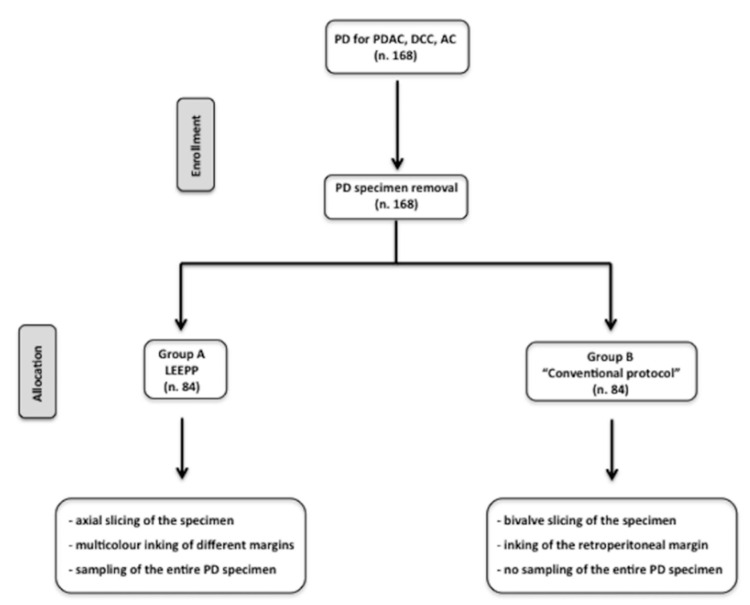
PRISMA flow-chart showing randomization process for the pathological evaluation of PD specimen: Group A (LEEP) and Group B (conventional protocol).

After PD was performed, a computer-generated randomization was used to allocate PD specimens to two different methods of pathological evaluation: the LEEPP (group A) or a conventional protocol which was already used by each participating center (group B) (Figure 1).

**Figure 2:**
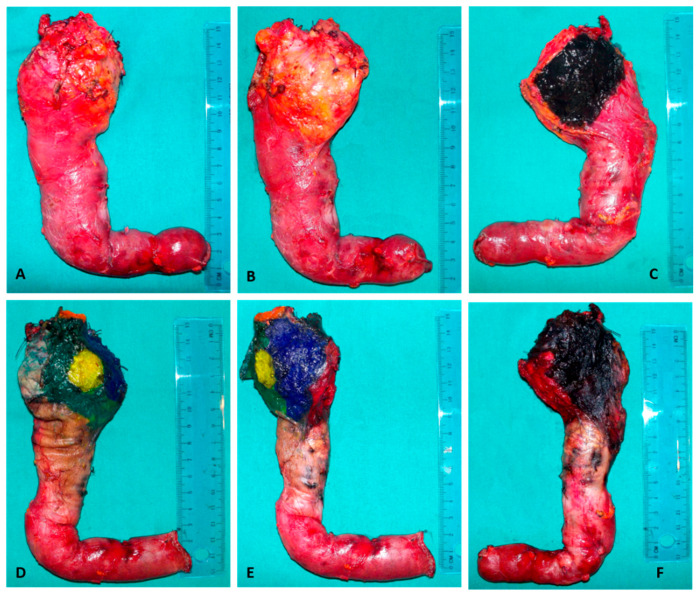
Evaluation of resection margins in PD specimens. (**A**–**C**) Pancreaticoduodenectomy specimen in Group B (“conventional protocol): (**A**) View of anterior surface. (**B**) View of medial surface. (**C**) View of posterior surface: the posterior margin (variably defined as uncinate margin or retroperitoneal margin) is inked in black. (**D**–**F**) Pancreaticoduodenectomy specimen in Group A (LEEPP protocol). The resection margins are painted in different colors: (**D**) View of anterior surface. The anterior margin is inked in green. (**E**) View of medial surface. The medial margin includes superior mesenteric vein margin inked in blue and superior mesenteric artery margin inked in red. In this image is also visible the pancreatic stumps inked in yellow and the bile duct stump in orange. (**F**) View of posterior surface painted in black.

LEEPP has previously been well described in the literature [7]. The main features of the protocol are axial slicing of the specimen, multicolor margin staining, and sampling of the entire specimen [7]. For group B, PD specimens underwent gross anatomical examination according to Rosai-Ackerman’s Surgical Pathology Textbook [12]. This protocol includes the evaluation of all anatomic structures (pancreatic duct, ampulla of Vater, common bile duct, and pancreatic head), without the inclusion of the entire specimen and bivalve slicing of the specimen [12] (Figure 2).

Each participating center, before enrolment for the study, practiced the LEEPP protocol in at least five patients.

### 2.2. Study End-Points and Data Collection

Our aims were to evaluate the effect of the pathological method (LEEPP vs. conventional) and the definition of clearance (0 vs. 1 mm) in terms of R1 resection rate and to assess with which pathological method and clearance R1 resection can be considered a significant prognostic factor.

The following clinical and pathological variables were systematically collected: patient demographics; neoadjuvant and/or adjuvant treatments; operative data (Whipple/Pylorus-Preserving-PD [PPPD], vascular resections); number of paraffin-embedded blocks; tumor histology (pancreatic, ampullary or biliary cancer); tumor grade (G); tumor size and spread (pT); perineural invasion; lymphatic invasion; vascular invasion; number of retrieved lymph nodes from the specimen; total number of harvested lymph nodes (lymph nodes retrieved with the specimen + those analyzed separately from the specimen); and lymph node status (pN). Tumor histology was defined according to the WHO classification of tumors [19]. Staging was assessed according the pTNM of American Joint Committee on Cancer (AJCC) [20]. The R1 rate was calculated adopting two clearances (0 and 1 mm) for both protocol groups. A sub-analysis of R1 rate according to different tumor histology (PDAC, DCC, AC) was also performed (Table S1). The median time consumption for the pathological analysis (macroscopic evaluation, slide preparation, and microscopic analysis) was calculated for each study group.

All cases were evaluated during an institutional multidisciplinary tumor board conference, to discuss indications to surgery as well as to neo-/adjuvant treatments. The optimal chemotherapy regimen was selected based on tumor histology and according to international guidelines at the time of treatment [21,22].

Follow-up was conducted according to a standardized schedule (1 month after surgery, then every 4–6 months for the first 5 years). Local recurrence (LR) was defined as the radiological re-appearance of the disease at the site of surgery (peri-anastomotic or nodal invasion). Overall survival (OS) was defined by the time of death after surgery.

### 2.3. Sample Size Calculation and Statistical Analysis

The sample size calculation was based on the assumption of at least a 2-fold increase in the R1 rate with the adoption of LEEPP, as indicated in the available literature [6].

Given a 30% incidence of R1 at the coordinating center before the beginning of the study, we expected an R1 rate of at least 60% of cases. Alpha was set at 0.05, and the power was set at 80%, suggesting a total study population of 128 patients. Forty more patients were added as a controlling factor due to possible confounding effects.

The χ^2^ test (using Yates’ correction when the sample size was greater than 40) or Student’s t test (after verifying normality of the data distribution using the Shapiro–Wilk test) were used to assess differences in clinical data between groups.

To assess risk modification of the LR between groups, a logistic regression model was used.

The survival data were analyzed using Kaplan–Meier analysis, the log-rank test and Cox regression. The results are expressed as the probability (*p*) that the null hypothesis (no difference exists) is true.

Values of *p* less than 0.05 were considered significant. In order to evaluate the prognostic role of R1 resection, different multivariate analysis was performed according to the method of pathological evaluation (group A and B) and to the minimum clearance definition (0 and 1 mm).

Multivariate analyses according to the method of pathological evaluation and clearance were also performed, in order to identify significant prognostic factors.

## 3. Results

Between March 2013 and June 2015, 168 patients were enrolled, and their PD specimens included in the study. The clinical and histopathological data of both groups are reported in Table 1.

The two groups were similar in terms of pre-operative and intra-operative data. The adoption of the LEEPP significantly increased the number of examined blocks (49.8 vs. 35.9; *p* < 0.01) and the number of retrieved lymph nodes (39.7 vs. 29.5; *p* = 0.01). Pathological data (grading, nodal involvement, perineural and lympho-vascular invasion) did not significantly differ between the two groups, except for tumor size (2.67 (0.4–5.5) vs. 2.97 (1–7) for group A and B, respectively) (*p* = 0.04). Even if did not reach a statistical significance, tumor origin was different in the two groups (PDAC 66% vs. 81%, AC 23% vs. 10%, DC 11% vs. 8%, for group A and B, respectively; *p* = 0.06).

### 3.1. Impact of Method of Pathological Evaluation and Clearance on R1 Rate

Table 2 reports the R1 rate for each group using the two definitions of clearance. A significant increase in R1 rate was observed in each group when using a definition of 1 mm rather than 0 mm (60.7% versus 26.2% in group A and 57.1% versus 20.2% in group B; both *p* < 0.05). No significant differences in R1 rate were observed between the two groups if the same clearance definition was used (26.2% vs. 20.2% with 0 mm (*p* = 0.36), and 60.7% vs. 57.1% with 1 mm for groups A and B, respectively) (*p* = 0.27)). Similar results were obtained for each tumor histology (Table 2).

**Figure 3:**
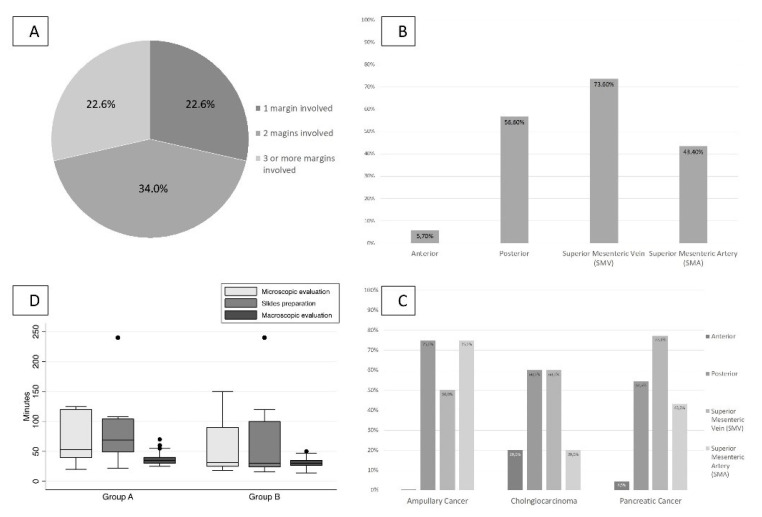
(**A**). Frequency of involved margins in R1 cases. (**B**). Rate of microscopic invasion for each margin in R1 cases. (**C**). Rate of microscopic invasion for each margin in R1 cases for each periampullary tumor. (**D**). Time consumption for the pathological evaluation.

The use of a multicolor inking in the LEEPP showed that more than one margin was involved in 56.6% of R1 resections (Figure 3A), with superior mesenteric vein (SMV) the most frequently involved margin (73.6%) (Figure 3B). Figure 3C shows the margin involvement according to tumor histology.

The time consumption of pathologic analysis (Figure 3D) was significantly higher for group A than for group B (macroscopic evaluation, 36 vs. 30 min; slide preparation, 104 vs. 82 min; microscopic evaluation, 70 vs. 50 min) (*p* < 0.05).

### 3.2. Prognostic Role of R1 Resection

The median follow-up of the entire cohort was 34 months. LR was observed in 44 patients (26.2%), 22 in each study group. Regardless of the pathological protocol and clearance definition, R1 rate was not significantly associated with an increase in LR (*p* > 0.05) (Table 3).

**Figure 4:**
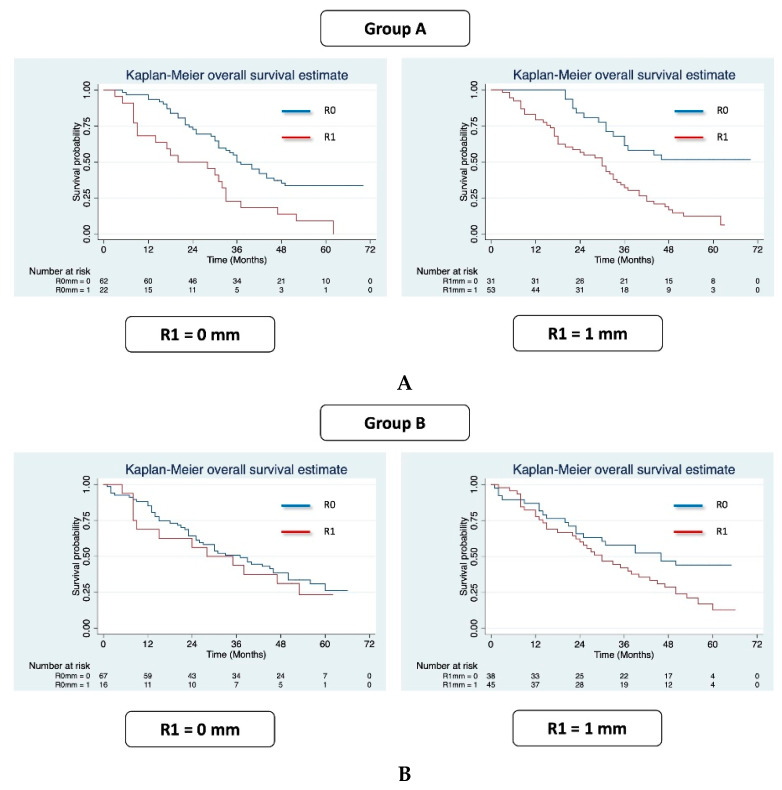
(**A**) Kaplan-Meier curves according to R status. OS for group A at 0 mm (**left side**) and 1 mm (**right side**) clearance. (**B**) Kaplan-Meyer curves according to R status. OS for group B at 0 mm (**left side**) and 1 mm (**right side**) clearance.

Figure 4A shows the OS Kaplan–Meier curves according to R status for each study group and for each clearance (0 and 1 mm). For group A, the median OS was 36 (31–46) months for R0 and 20 (9–33) months for R1 (HR 2.35; *p* = 0.007) using 0 mm clearance. When adopting 1 mm clearance, the median OS was not reached for R0 and was 30 (18–34) months (HR 3.55; *p* = 0.005) for R1 (Figure 4A).

For group B, when adopting 0 mm clearance, the median OS was 37 (25–46) and 28 (8–53) months (HR 0.82; *p* = 0.607) for R0 and R1, respectively, while using 1 mm clearance, median OS was 46 (23 not reached) for R0 and 30 (22–40) months for R1 (HR 1.66; *p* = 0.142) (Figure 4B).

Table 4A,B shows multivariate analyses according to the method of pathological evaluation (groups A and B) and minimum clearance (0 and 1 mm). For group A, R1 resection was a significant prognostic factor independent of clearance (HR 2.19 (1.20–4.00), *p* = 0.011, at 0 mm; HR 3.34 (1.40–8.00), *p* = 0.007, at 1 mm) (Table 4A). Conversely, for group B, R1 resection independent of clearance was not a significant prognostic factor (HR 0.88 (0.43–1.80), *p* = 0.729, at 0 mm; HR 1.96 (0.96–4.01), *p* = 0.066, at 1 mm) (Table 4B).

## 4. Discussion

Since the introduction of LEEPP by Verbeke et al. in 2006 [6], it has been clarified that two main pathological aspects influence R1 resection rate after PD for periampullary cancer: the method of pathological evaluation, in terms of slicing and sampling of PD specimen, and the definition of minimum clearance (0 or 1 mm). However, the available literature is extremely heterogeneous, with some studies adopting LEEPP with a 1 mm clearance definition [10,23], others using 1 mm clearance but with a different sampling of PD specimens (bivalve slicing, evaluation only of the retroperitoneal margin) [24], and others using 0 mm clearance [25,26,27]. This heterogeneity prevents the correct comparison and interpretation of available results. In 2015, the International Study Group of Pancreatic Surgery (ISGPS) recommended the use of the 1 mm clearance and a consensus of clearance definition was reached [28]

For this reason, we conducted this multicenter randomized study, which compared the LEEPP with the conventional method used by the participating centers before the beginning of the study. Our first aim was to evaluate the specific impact of the pathological evaluation protocol and definition of clearance in determining R1 rate after PD for periampullary cancer. This was possible because, unlike previously published studies [6,10], we utilized both clearance definitions (0 and 1 mm) for both groups. Our results confirmed that the clearance definition used, more than the kind of pathological evaluation, significantly affects R1 rate independent of the method of pathological evaluation.

By shifting from 0 to 1 mm, R1 significantly increased from 26.2% to 60.7% and from 20.2% to 57.1% for groups A and B, respectively (*p* < 0.05); on the other hand, adopting the same clearance definition, no difference in terms of R1 rate was found between the two groups (26.2% vs. 20.2% at 0 mm, 60.7% vs. 57.1% at 1 mm, for group A and B, respectively; *p* > 0.05). This result confirmed those reported by previous studies, which showed that retrospectively adopting 1 mm instead of 0 mm clearance resulted in a significant increase in the R1 resection rate [15,29]. Moreover, the insignificant impact of the kind of pathological evaluation in terms of R1 rate reported in our study confirmed that reported by a recent randomized controlled trial published by the Dutch Pancreatic Cancer Group. In this study, bivalve and axial slicing of PD specimen were evaluated: the R1 rate was similar with the two slicing techniques (60% and 55%, respectively; *p* = 0.71) [30].

However, the adoption of the LEEPP did result in significant differences in terms of other pathological findings, such as a higher number of paraffin blocks and of retrieved lymph nodes. An increased number of positive lymph nodes was also observed, although this was not statistically significant. These results, as also reported in other studies [6,10,31], could be explained by the inclusion of the entire PD specimen with the LEEPP. However, although LEEPP is more accurate, it is also more demanding for the pathologist. In our study, LEEPP required more time for macroscopic evaluation, slide preparation, and microscopic evaluation compared with the conventional method.

Aside from the reported rate of R1 resection after PD, the most important aspect to take into consideration is the impact of R1 on prognosis. This aspect is still debated, with some studies but not others reporting a prognostic impact of R1 resection [14,15,16,17,18]. One explanation for this heterogeneity is the lack of the above mentioned consensus. The most important aim of the current study was to identify the method of PD sampling and clearance that best identify R1 resections associated with worse prognosis. We demonstrated that, independent of the adopted clearance, LEEPP correctly identified the R1 resections that affected prognosis (median OS: 36 vs. 20 months for R0 and R1 at 1 mm clearance; not reached vs. 30 months for R0 and R1 at 1 mm clearance). Conversely, using the conventional method of pathological evaluation, no significant association between R1 and OS was found independent of clearance (median OS: 37 vs. 28 months for R0 and R1 at 0 mm clearance; 46 vs. 30 months for R0 and R1 at 1 mm clearance). This result was confirmed with multivariate analyses, which showed that, independent from the adopted clearance, R1 resection was a significant prognostic factor only when LEEPP was used. Our hypothesis is that the evaluation of the entire PD specimen in LEEPP determines a more accurate pathological evaluation and better identifies the true R1 resections, able to significantly affect prognosis.

Moreover, we evaluated the relationship between R1 and the development of LR. Some studies have reported that R1 is a predictive factor for LR [32,33]. However, our results indicated that R1 resection was not significantly associated with an increased risk of LR. Interestingly, this lack of association was seen independently from the adopted pathological method or clearance. Possibly, rather than being a good predictive factor for LR, R1 maybe an effective indicator of more aggressive biological behaviour of the tumor.

One of the strengths of this study was its randomized fashion, in that specimens rather than patients were randomized. In our opinion, even if the randomization does not take into account any intervention, this is important because the allocation during the same study period allowed the two groups to be more homogeneous in terms of treatment (surgical strategy and adjuvant treatment). Another strength of the study was the long follow-up time (median follow-up 34 months).

This study has some limitations. Firstly, we included all periampullary cancers, even if our series was composed mostly of PDAC (>70%). Further studies evaluating each specific periampullary cancer are needed. The second limitation was that group B could not be completely homogeneous and differences in the conventional method used between the participating centers may have been present. However, the two most significant aspects of the pathological evaluation (bivalve slicing technique and the absence of the inclusion of the entire specimen) were adopted by each center.

## 5. Conclusions

The clearance definition (0 or 1 mm), independent of the pathological method, is the most important factor affecting R1 rate after PD for periampullary cancer. The pathological method used is crucial to accurately evaluate the prognostic role of R1 resections, with R1 only a prognostic factor independent of clearance with the adoption of LEEPP. A worldwide consensus on adopted pathological protocol and clearance is urgently needed.

## Figures and Tables

**Table 1:** Demographic, clinical, and histopathological data.

	LEEPP (Group A) (*N*. 84)	“Conventional” Protocol (Group B) (*N*. 84)	*p*
Gender:			0.21
(a) male, *n*. (%)	48 (57%)	40 (52%)	
(b) female, *n*. (%)	36 (42%)	44 (47%)	
Age, mean years (range)	67.5 (33–84)	67.3 (38–85)	0.87
Neo-adjuvant treatment, *n*. (%)	7 (8.3%)	5 (5.9%)	0.38
Type of surgery:			0.83
(a) Classical Whipple, *n*. (%)	13 (15%)	14 (17%)	
(b) PPPD, *n*. (%)	71 (85%)	70 (83%)	
Vascular resection, *n*. (%)	16 (19%)	17 (20%)	0.84
Histological type:			0.06
(a) PDAC, *n*. (%)	55 (66%)	68 (81%)	
(b) AC, *n*. (%)	20 (23%)	9 (10%)	
(c) DC, *n*. (%)	9 (11%)	7 (8%)	
Histological grade:			0.62
(a) Gx, *n*. (%)	6 (7.14%)	6 (7.14%)	
(b) G1, *n*. (%)	2 (2.38%)	6 (7.14%)	
(c) G2, *n*. (%)	40 (47.62%)	39 (46.43%)	
(d) G3, *n*. (%)	35 (41.67%)	31 (36.90%)	
(e) G4, *n*. (%)	1 (1.19%)	2 (2.38%)	
Tumor size, mean cm (range)	2.67 (0.4–5.5)	2.97 (1–7)	0.04
T staging:			0.12
(a) Tis, *n*. (%)	1 (1.2%)	0 (0%)	
(b) T1, *n*. (%)	2 (2.4%)	2 (3.6%)	
(c) T2, *n*. (%)	10 (11.9%)	2 (2.4%)	
(d) T3, *n*. (%)	65 (77.5%)	75 (88.1%)	
(e) T4, *n*. (%)	6 (7.1%)	5 (5.9%)	
N staging:			0.25
(a) N0, *n*. (%)	15 (17.9%)	21 (25.0%)	
(b) N+, *n*. (%)	69 (82.1%)	63 (75.0%)	
Vascular invasion, *n*. (%)	44 (52.4%)	48 (57.1%)	0.53
Lymphatic invasion, *n*. (%)	32 (38.1%)	32 (38.1%)	0.99
Perineural invasion, *n*. (%)	67 (79.8%)	66 (78.6%)	0.84
N. of retrieved LN, mean (range)	39.7 (12–97)	29.5 (5–97)	0.01
N. of metastatic LN, mean (range)	4.7 (0–29)	4.3 (0–25)	0.71
N. of blocks, mean (range)	49.8 (20–95)	35.9 (13–109)	<0.01
Adjuvant treatment, *n*. (%)	67 (79.7%)	58 (69.8%)	0.12

PPPD: Pylorus-preserving Pancreaticoduodenectomy; PDAC: Pancreatic Ductal Adenocarcinoma; DC: distal cholangiocarcinoma; AC: ampullary cancer; LN: lymphnode.

**Table 2:** R1 resection rate in both study’s groups, adopting 0 mm and 1 mm clearance.

Minimum Clearance	R1 Resection (%) (Group A)	R1 Resection (%) (Group B)	*p*
Entire cohort:			
(a) 0 mm, *n*. (%)	22 (26.2%)	17 (20.2%)	0.36
(b) 1 mm, *n*. (%)	51 (60.7%)	48 (57.1%)	0.27
PDAC *			
(a) 0 mm, *n*. (%)	18 (32.7%)	16 (23.5%)	0.25
(b) 1 mm, *n*. (%)	44 (80.0%)	43 (63.2%)	0.42
DC:			
(a) 0 mm, *n*. (%)	1 (11.1%)	1 (14.3%)	0.84
(b) 1 mm, *n*. (%)	5 (55.5%)	1 (14.3%)	0.09
AC:			
(a) 0 mm, *n*. (%)	3 (15.0%)	0 (0%)	0.22
(b) 1 mm, *n*. (%)	2 (20.0%)	4 (22.2%)	0.89

* PDAC: Pancreatic Ductal Adenocarcinoma; DC: distal cholangiocarcinoma; AC: ampullary carcinoma.

**Table 3:** Association between LR and R1 resection in both study groups, according 0 mm and 1 mm clearance.

	Group A			Group B		
	R0 Resection	R1 Resection	*p*	R0 Resection	R1 Resection	*p*
LR * (0 mm), *n*. (%)	15 (24.2%)	7 (31.8%)	0.48	18 (26.9%)	4 (25.0%)	0.87
LR * (1 mm), *n*. (%)	6 (19.2%)	16 (30.1%)	0.27	11 (28.9%)	11 (24.4%)	0.64

* LR: Local recurrence.

**Table 4:** Multivariate analysis of prognostic factors according to method of pathological evaluation and minimum clearance.

**A.** Multivariate analyses for group A according to the different minimum clearance				
	**Group A** **(R1 Clearance = 0 mm)**		**Group A** **(R1 Clearance = 1 mm)**	
	**HR**	***p*-Value**	**HR**	***p*-Value**
Age	1.00 (0.97–1.04)	0.709	0.99 (0.96–1.03)	0.749
Sex female	1.33 (0.78–2.27)	0.297	1.44 (0.82–1.53)	0.197
PDAC vs.:				
DCC	0.61 (0.22–1.68)	0.340	0.72 (0.26–1.99)	0.528
AC	0.44 (0.17–1.16)	0.099	0.67 (0.24–1.79)	0.421
Neoadjuvant treatment	0.31 (0.08–1.15)	0.080	0.27 (0.78–1.12)	0.074
Adjuvant treatment	0.15 (0.06–0.39)	0.0001	0.17 (0.06–0.42)	0.0001
pT3–pT4	1.69 (0.26–10.99)	0.581	1.70 (0.28–10.47)	0.568
N+	2.08 (0.93–4.68)	0.076	1.62 (0.73–3.59)	0.237
G3–G4	1.09 (0.58–2.06)	0.779	1.34 (0.72–2.51)	0.356
Vascular invasion	2.16 (1.16–4.03)	0.015	1.99 (1.06–3.71)	0.031
Perineural invasion	1.31 (0.23–7.52)	0.762	0.92 (0.16–5.32)	0.929
R1	2.19 (1.20–4.00)	0.011	3.35 (1.40–8.00)	0.007
**B.** Multivariate analyses for group B according to the different minimum clearance.				
	**Group B** **(R1 Clearance) = 0 mm**		**Group B** **(R1 Clearance) = 1 mm**	
	**HR**	***p*-Value**	**HR**	***p*-Value**
Age	1.02 (0.99–1.05)	0.170	1.02 (0.99–1.05)	0.223
Sex female	1.89 (1.03–3.46)	0.039	1.88 (1.04–3.40)	0.037
PDAC vs.:				
DCC	1.25 (0.44–3.55)	0.672	1.81 (0.59–5.51)	0.297
AC	0.13 (0.03–0.51)	0.003	0.19 (0.05–0.78)	0.021
Neoadjuvant treatment	1.47 (0.38–5.58)	0.574	1.68 (0.44–6.42)	0.760
Adjuvant treatment	0.21 (0.11–0.43)	0.0001	0.21 (0.10–0.42)	0.0001
pT3–pT4	0.80 (0.10–6.39)	0.838	1.05 (0.13–8.25)	0.963
N+	1.86 (0.66–5.17)	0.238	1.53 (0.56–4.20)	0.407
G3–G4	1.11 (0.56–2.21)	0.766	0.97 (0.49–1.90)	0.929
Vascular invasion	1.51 (0.77–2.98)	0.227	1.34 (0.68–2.64)	0.392
Perineural invasion	0.41 (0.17–0.97)	0.044	0.39 (0.16–0.95)	0.039
R1	0.88 (0.43–1.80)	0.729	1.96 (0.96–4.01)	0.066

## Data Availability

The data presented in this study are available in this article.

## Supplementary Materials

The following are available online at https://www.mdpi.com/article/10.3390/cancers13092097/s1, Table S1: Histopathological results according to tumor histology.
