# Supplementary material for: The Role of Pathological Method and Clearance Definition for the Evaluation of Margin Status after Pancreatoduodenectomy for Periampullary Cancer. Results of a Multicenter Prospective Randomized Trial
Source: Cancers (Basel). 2021 Apr 26;13(9):2097. doi: 10.3390/cancers13092097 (PMC8123600; doi:10.3390/cancers13092097)
Supplement: Supplementary file 1 [file cancers-13-02097-s001.zip › cancers-1168077-supplementary.pdf]

Table S1. Histopathological results according to tumor histology.

	PDAC (N. 123)			Ampullary cancer (N. 29)			Distal cholangioK (N. 16)		
	**Group A (N. 55)**	**Group B (N. 68)**	**P**	**Group A (N. 20)**	**Group B (N. 9)**	**p**	**Group A (N. 9)**	**Group B (N. 7)**	**p**
Tumor size, main cm (range)	3.09 (3.0–3.2)	3.11 (3.0–3.2)	> 0.05	2 (1.8–2.2)	2.2 (1.9–2.5)	> 0.05	1.57 (1.3–1.8)	2.6 (2.1–3)	**< 0.05**
T status:<br>- Tis, *n*. (%)<br>- T1, *n*. (%)<br>- T2, *n*. (%)<br>- T3, *n*. (%)<br>- T4, *n*. (%)	 0 (0%)<br>0 (0%)<br>0 (0%)<br>53 (96.4%)<br>2 (3.6%)	 0 (0%)<br>0 (1.5%)<br>0 (0%)<br>67 (97.0%)<br>1 (1.5%)	> 0.05	 0 (0%)<br>2 (10%)<br>10 (50%)<br>4 (20%)<br>4 (20%)	 0 (0%)<br>1 (11.1%)<br>2 (22.2%)<br>2 (22.2%)<br>4 (44.4%)	> 0.05	 1 (11.1%)<br>0 (%)<br>0 (0%)<br>8 (88.9%)<br>0 (0%)	 0 (0%)<br>1 (14.3%)<br>0 (0%)<br>6 (85.7%)<br>0 (0%)	> 0.05
Nodal involvement, *n*. (%)	48 (87.2%)	53 (77.9%)	> 0.05	14 (70.0%)	4 (44.4%)	> 0.05	7 (77.7%)	6 (85.7%)	> 0.05
Grading:<br>- Gx, *n*. (%)<br>- G1, *n*. (%)<br>- G2, *n*. (%)<br>- G3, *n*. (%)<br>- G4, *n*. (%)	 5 (9.1%)<br>0 (0%)<br>29 (52.7%)<br>20 (36.4%)<br>1 (1.9%)	 6 (8.8%)<br>2 (2.9%)<br>35 (51.5%)<br>24 (35.3%)<br>6 (8.8%)	> 0.05	 0 (0%)<br>1 (5%)<br>6 (30%)<br>13 (65%)<br>0 (0%)	 0 (0%)<br>3 (33.3%)<br>2 (22.2%)<br>4 (44.4%)<br>0 (0%)	> 0.05	 0 (0%)<br>2 (22.2%)<br>5 (55.5%)<br>2 (22.2%)<br>0 (0%)	 0 (0%)<br>1 (14.3%)<br>2 (28.6%)<br>3 (42.0%)<br>1 (14.3%)	> 0.05
Vascular invasion, *n*. (%)	29 (52.7%)	41 (60.2%)	> 0.05	12 (60%)	4 (44.4%)	> 0.05	3 (33.3%)	3 (42.8%)	> 0.05
Lymphatic invasion, *n*. (%)	21 (38.1%)	25 (36.7%)	> 0.05	9 (45.0%)	3 (33.3%)	> 0.05	2 (22.2%)	4 (57.4%)	> 0.05
Perineural invasion, *n*. (%)	53 (96.3%)	59 (86.7%)	> 0.05	7 (35.0%)	2 (22.2%)	> 0.05	7 (77.7%)	5 (71.4%)	> 0.05
N. of retrieved LN*, mean (range)	43.1 (12–97)	30.6 (5–97)	**<.001**	32.5 (17–50)	24.7 (14–39)	**< 0.05**	34.7 (21–48)	25.8 (18–38)	**< 0.05**
N. of metastatic LN*, mean (range)	5.5 (0–29)	4.9 (0–25)	> 0.05	3.6 (0–16)	0.8 (0–3)	> 0.05	1.8 (0–4)	3.7 (0–12)	> 0.05
N. of blocks, mean (range)	49.2 (20–95)	37.0 (13–109)	**< 0.01**	49 (25–77)	29.6 (15–70)	**< 0.01**	53.8 (33–70)	34 (16–83)	**< 0.05**
Adjuvant	47 (85.4%)	49 (73.1%)	>	13 (65.0%)	2 (22.2%)	<	7 (77.7%)	7 (100%)	> 0.05

treatment, *n*. (%)			0.05			**0.05**			

*LN: lymphnode
